# Draft genome sequence of *Aeromonas veronii* strain Naf-IIUM T8S isolated from diseased *Pangasianodon hypophthalmus* in Malaysia

**DOI:** 10.1128/mra.00407-26

**Published:** 2026-08-24

**Authors:** Nadia-Sabrina Afandi, Najatul Su-Ad Abdullah, Rimatulhana Ramly, Mohd Firdaus-Nawi, Azila Abdullah, Nur-Nazifah Mansor, Muhamad Izzuan-Razali, Zahin Azhari Zairi-Noor, Yulianna Puspitasari

**Affiliations:** 1 Aquatic Health Research Laboratory, Institute of Oceanography and Maritime Studies, Kulliyyah of Science, International Islamic University Malaysia, Kuantan, Pahang, Malaysia; 2 Department of Fisheries, National Fish Health Research Centre, Fisheries Research Institute, Batu Maung, Penang, Malaysia; 3 Division of Veterinary Microbiology, Faculty of Veterinary Medicine, Universitas Airlangga, Surabaya, Indonesia; DOE Joint Genome Institute, Berkeley, California, USA

**Keywords:** *Aeromonas veronii*, motile Aeromonas Septicemia (MAS), genome, Naf-IIUM T8S, Pahang River, *Pangasianodon hypopththalmus*, AMR genes

## Abstract

The draft genome of *Aeromonas veronii* strain Naf-IIUM T8S isolated from a diseased iridescent shark cultured in the Pahang River, Malaysia. The genome comprises three contigs with a total length of 4,797,547 bp, encoding 4,517 genes and a GC content of 59%.

## ANNOUNCEMENT

*Aeromonas veronii* is an opportunistic gram-negative bacterium and a causative agent of motile Aeromonas Septicemia (MAS), affecting freshwater fish worldwide (1–6). Here, *A. veronii* strain Naf-IIUM T8S was isolated from a cage-cultured iridescent shark (*Pangasianodon hypophthalmus*) in the Pahang River, Malaysia. The fish exhibited clinical signs consistent with MAS. The infected specimen was obtained from a farm (3°43′39.12″ N, 102°22′16.83″ E) on 1st October 2025. Spleen tissues were streaked onto tryptic soy agar plates and incubated at 30°C for 24 h under aerobic conditions. Colonies with round, creamy morphology were repeatedly subcultured to obtain a pure isolate. Genomic DNA was extracted from pooled bacterial colonies using the PrimeWay Genomic II DNA Extraction Kit (1st Base-Apical Scientific, Malaysia). DNA quality was assessed with NanoDrop Lite spectrophotometer (Thermo Fisher Scientific, USA) and 1.2% agarose gel electrophoresis. Preliminary identification was performed by the amplification of the 16S rRNA gene using genus-specific primer (Genbank accession no. PX953912.1).

Genomic DNA libraries were prepared using the Oxford Nanopore Technologies (Oxford, UK) Ligation Sequencing gDNA Native Barcoding Kit 24 v14 (SQK-NBD114.24) following the manufacturer’s protocol without DNA shearing and size-selection. Sequencing was conducted on a MinION Mk1D device with an R10.4.1 flow cell (FLO-MIN114) using MinKNOW v.6.5.15. Basecalling, demultiplexing, and barcode trimming were conducted using Guppy with default parameters. A total of 226,605 reads were generated (mean length, 5,287.9 bp; median, 3,033 bp; read N_50_ 8,867 bp; mean quality score; 16.3). Reads were processed using the EPI2ME bacterial genomes workflow v.1.4.2, filtered with fastcat v.0.22.0. *De novo* assembly was done using Flye v.2.9.5 (7) (~250× coverage), followed by polishing with Medaka v.2.0.0, and annotation with Prokka v.1.14.5 (8). The draft genome comprises three contigs (4,602,237 bp, 113,037 bp, and 82,134 bp) with an overall size of 4,797,547 bp (N_50_ = 4,602,237 bp), GC content of 59%, and 4,517 predicted genes. Genome completeness was estimated at 100% using CheckM2 v.1.1.0 (9) via Galaxy Server v.25.1.2.dev0 (10, 11) with default parameters. This isolate was identified as *A. veronii* based on *gyrB* gene analysis using BLAST (12), which returned a best hit to *A. veronii* strain HS1906 (accession no. CP133079.1), revealing 100% query cover and identity.

**Fig 1:**
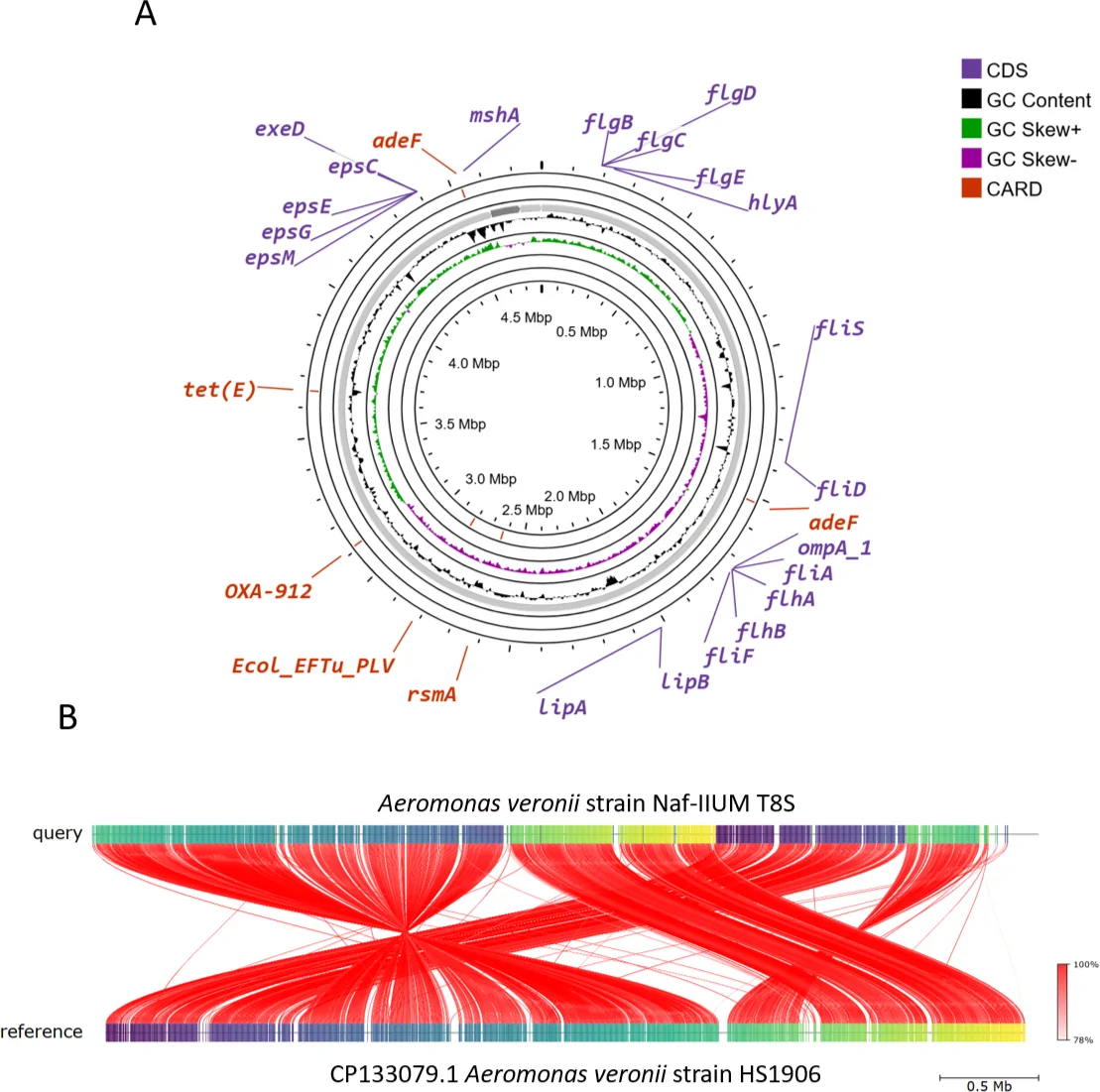
(**A**) The draft genome of *A. veronii* strain Naf-IIUM T8S visualized using Proksee, exhibiting the virulence genes (purple) and antimicrobial resistance genes (red) identified using VFDB and CARD RGI. (**B**) Whole-genome alignment between the present isolate and reference (*A. veronii* strain HS1906; CP133079.1). Red links indicate homologous regions between the query (top) and reference (bottom) genomes, with color intensity representing nucleotide identity (78%–100%). Scale bar = 0.5 Mb.

Virulence genes were identified by comparing against Virulence Factor Database v.6.0 (13, 14) and supported by literature (2, 5, 6). Genome comparison using FastANI v.1.2.0 (15) in Proksee (16) displayed 98.06% average nucleotide identity to *A. veronii* strain HS1906, with 1,371 out of 1,598 orthologous matches (Fig. 1). CARD Resistance Gene Identifier (RGI) v.1.4.1 (17) in Proksee was used to identify AMR genes, (*adef*, *OXA-912*, *Ecol_EFTu_PLV*, and *rmsA*), consistent with previous studies (1, 3, 18).

## Data Availability

All data, including raw reads, are available at NCBI Sequence Read Archive with accession number SRR38918174. The genome assembly of *A.veronii* strain Naf-IIUM T8S can be accessed under accession number JBVNBL000000000.1.
